# Inhibition of *Listeria monocytogenes* Growth, Adherence and Invasion in Caco-2 Cells by Potential Probiotic Lactic Acid Bacteria Isolated from Fecal Samples of Healthy Neonates

**DOI:** 10.3390/microorganisms11020363

**Published:** 2023-01-31

**Authors:** Sofia V. Poimenidou, Athina Skarveli, Georgia Saxami, Evdokia K. Mitsou, Maria Kotsou, Adamantini Kyriacou

**Affiliations:** Laboratory of Biology, Biochemistry, Physiology and Microbiology, Department of Nutrition and Dietetics, School of Health Science and Education, Harokopio University of Athens, 17671 Athens, Greece

**Keywords:** gut microbiota, *Lactobacillus*, pathogens, epithelial cells, competition, exclusion, displacement, pre-treatment

## Abstract

Lactic acid bacteria (LAB) isolated from healthy humans may prove an effective tool against pathogen growth, adherence and invasion in intestinal epithelial cells. This study aimed to evaluate the antilisterial properties of LAB isolated from fecal samples of healthy neonates. Forty-five LAB strains were tested for their antimicrobial activity against ten *Listeria monocytogenes* strains with spot-on-lawn and agar-well diffusion assays, and ten lactobacilli strains were further assessed for their inhibitory effect against adherence and invasion of Caco-2 cells by *L. monocytogenes* EGDe. Inhibition was estimated in competition, exclusion or displacement assays, where lactobacilli and *L. monocytogenes* were added to Caco-2 monolayers simultaneously or 1 h apart from each other. Inhibition of *L. monocytogenes* growth was only displayed with the spot-on-lawn assay; cell-free supernatants of lactobacilli were not effective against the pathogen. *Lactobacillus (L.) paragasseri* LDD-C1 and *L. crispatus* LCR-A21 were able to adhere to Caco-2 cells at significantly higher levels than the reference strain *L. rhamnosus* GG. The adherence of *L. monocytogenes* to Caco-2 cells was reduced by 20.8% to 62.1% and invasion by 33.5% to 63.1% during competition, which was more effective compared to the exclusion and displacement assays. These findings demonstrate that lactobacilli isolated from neonatal feces could be considered a good candidate against *L. monocytogenes*.

## 1. Introduction

The gastrointestinal (GI) microbiota of healthy humans consists of bacteria, archaea, fungi and viruses that coexist in a mutualistic association with the host, a condition described as eubiosis [1,2]. Gut microbiota contributes to human health, where besides food digestion, it also regulates nutrient metabolism, stimulates the immune system, maintains the integrity of the mucosal barrier and protects the host against pathogens [3]. The microbial colonization of the human gut begins at birth and takes almost three years to reach the complexity and diversity of the adult’s gut microbiota [4]. The colonizing bacteria are derived from the mother, breast milk and surrounding environment, and the colonization is influenced by extrinsic and intrinsic factors, which include geographic area, mode of delivery, feeding habits and genetics [5]. The microbial balance can be disturbed by environmental factors, lifestyle or diseases, resulting in ratio alterations of beneficial and potentially harmful microorganisms that can lead to dysbiosis [6]. Probiotics are living microbial strains that are able to exclude or inhibit pathogens and restore the balance in the GI tract to enhance the function of the intestinal epithelial barrier and to modulate host immune responses [7,8].

Probiotics are mainly selected from the *Lactobacillus (L.)* and *Bifidobacterium (B.)* genera, and most of the currently used strains have been isolated from the intestinal microbiota of healthy humans [9]. When administered to the host, the probiotics need to survive passage through the stomach, be resistant to bile salts and digestive enzymes and reach the colon in sufficient numbers [10]. Lactic acid bacteria adhere to intestinal epithelial cells through passive forces, electrostatic interactions, hydrophobic and steric forces, lipotechoic acids and specific structures, and their colonization may prevent the adhesion of pathogenic bacteria to intestinal cells [11,12].

The competitive exclusion of pathogens by probiotics in the GI tract is mediated through diverse mechanisms. Probiotics compete with pathogens for limited nutrients, inhibit epithelial and mucosal adherence and invasion in epithelial cells, stimulate mucosal integrity and produce antimicrobial substances [12,13]. However, it has been established that there is a strain-specificity in probiotic properties and mechanisms, and a wide variation among isolates of the same species. Competition is dependent on both the pathogen and the probiotic strain, and therefore, a case-by-case study is needed for the probiotic selection [14,15,16].

*Listeria (L.) monocytogenes* is a foodborne pathogen, ubiquitous in nature, and able to survive hostile environments as a result of diverse adaptive mechanisms [17,18,19,20,21]. It is the causative agent of the foodborne illness listeriosis, which although rare, is characterized by a significant hospitalization and mortality rate. According to the European Food Safety Authority, 2183 cases of listeriosis were reported in the EU in 2021 with a 43.8% hospitalization rate and a 13.7% fatality rate [22]. In humans, listeriosis might occur as a mild noninvasive GI illness or as an invasive disease, and factors that are critical for the infection are related to the pathogen, host and the environment [23]. Previous studies have investigated the use of beneficial microbial cultures, such as *B. thermophilum*, *B. thermacidophilum, L. acidophilus*, *L. casei* and *L. rhamnosus*, as potential competitors against *L. monocytogenes* and highlighted the ability of probiotics to prevent infections by pathogens [24,25].

The objective of the present study was to investigate the inhibitory effect of lactobacilli isolates against *L. monocytogenes* growth, adherence and invasion in intestinal epithelial cells. The tested strains were isolated from healthy full-termed neonates and have been previously characterized for their probiotic attributes, which include acid and bile tolerance, adhesion ability to Caco-2 cells, antibiotic susceptibility and antimicrobial activity against the pathogens *Escherichia coli*, *Salmonella choleriasuis*, *L. monocytogenes*, *Enterococcus (E.) faecalis*, *E. hirrae* and *Staphylococcus aureus* [26,27]. Antimicrobial activity was estimated with the spot-on-lawn and the agar-well diffusion assays. Inhibition of adherence and invasion in epithelial cells were studied through competition, exclusion and displacement assays. The human colon adenocarcinoma Caco-2 cell line was used as a model of the intestinal barrier [28].

## 2. Materials and Methods

### 2.1. Bacterial Strains and Growth Conditions

Forty-three LAB strains, isolated from fecal samples of healthy full-term neonates from Greece, and two reference strains (Table 1) were tested for their antimicrobial capacity against ten different *L. monocytogenes* strains (Table 2). The LAB isolates belong to the collection of the Laboratory of Biology, Biochemistry, Physiology and Microbiology of Harokopio University of Athens, Greece, and have been previously studied for probiotic properties [26,27]. The *L. monocytogenes* strains were kindly provided by the Laboratory of Food Quality Control and Hygiene of Agricultural University of Athens, Greece, and have been previously characterized for virulence genes and biofilm formation capacity [18,29]. Prior to the experiments, the *L. monocytogenes* isolates were activated twice in tryptic soy broth (TSB; LabM, Lancashire, UK) supplemented with 0.6% yeast extract (YE; LabM) at 37 °C for 24 h in aerobic conditions (Memmert, Schwabach, Germany), and the lactobacilli were activated once on de Man, Rogosa and Sharpe agar (MRS; LabM) and twice in MRS broth at 37 °C under anaerobic conditions (BACTRON™ Anaerobic Chamber, Cornelius, OR, USA).

### 2.2. Antimicrobial Activity Assay

The antimicrobial activity of the LAB strains against *L. monocytogenes* was assessed with two methods: (i) the spot-on-lawn assay and (ii) the agar-well diffusion assay, according to Toure et al. [31]. In brief, for the spot-on-lawn assay, an aliquot of 3 μL of active MRS culture for each LAB strain was spotted on 45 mL MRS with 1.2% agar (Agar No2 Bacteriological, LabM) in a Petri dish (150 mm) and incubated anaerobically at 37 °C for 24 h. Following incubation, the Petri dishes were overlaid with 20 mL brain heart infusion (BHI; LabM) broth with 0.7% agar pre-warmed at 45 °C and inoculated with 0.3 mL of an overnight culture of *L. monocytogenes*. The Petri dishes were incubated aerobically for 24 h.

For the agar-well diffusion assay, activated LAB cultures were centrifuged at 3600 rpm for 10 min at 4 °C (Eppendorf Refrigerated Centrifuge, Hamburg, Germany), and the cell-free supernatant (CFS) of each LAB strain was adjusted to pH 6.5 with NaOH (4 M) and filtered through a 0.22 μm pore-size filter (Millex-GP Filter Unit, Merck Millipore Ltd., Cork, Ireland). Petri dishes (150 mm) that contained 20 mL solidified 1.2% agar were overlaid with 45 mL of BHI broth containing 0.7% agar and inoculated with 0.7 mL of an overnight *L. monocytogenes* culture. The plates were allowed to solidify for 1 h at ambient temperature (approx. 22 °C) and for 1 h at 4 °C. Wells of 5 mm diameter were cut in the solidified agar using a sterile metal-cork borer and filled with 40 μL of the CFS. The Petri dishes were incubated for 2 h at ambient temperature and for 16 h at 37 °C. For both methods, the presence of inhibitory zones around the spots or the agar wells were considered antimicrobial activity against *L. monocytogenes*. The antimicrobial assays were performed in duplicates.

### 2.3. Cell Culture

Enterocyte-like Caco-2 cells (ATCC^®^-HTB-37™) were cultured as monolayers in Dulbecco’s Modified Eagle’s medium (DMEM; PAN-Biotech GmbH, Aidenbach, Germany) supplemented with 10% (*v*/*v*) fetal bovine serum (FBS; Biochrom AG, Berlin, Germany) and 1% penicillin/streptomycin solution (10,000 U/mL, Biochrom AG). For the adhesion and invasion assays, Caco-2 cells were seeded at 10^5^ cells/mL/well in 24-well plates (TC-treated Cell Culture Plates, Flat Bottom, SPL Life Sciences Co. Ltd., Gyeonggi-do, Republic of Korea) in DMEM followed by 24 h starvation in DMEM supplemented with 0.1% FBS.

### 2.4. Adhesion Assay

Ten lactobacilli strains with strong adhesive abilities to Caco-2 cells [26] were selected for the competition assays against the *L. monocytogenes* EGDe strain. The adhesion assays were performed according to Moroni et al. [24]. Briefly, single overnight cultures of *L. monocytogenes* and lactobacilli were harvested by centrifugation at 3600 rpm for 10 min at 4 °C and washed twice with ¼-strength Ringer’s solution (LabM). The washed bacterial pellets were resuspended in DMEM with 0.1% FBS at a final concentration of ~10^6^ CFU/mL for *L. monocytogenes* EGDe and 10^6^–10^7^ CFU/mL for lactobacilli. Caco-2 cell monolayers developed in 24-well plates were inoculated with 500 μL of a single bacterial culture for the individual adhesion assays or with 250 μL of *L. monocytogenes* EGDe and 250 μL of each *Lactobacillus* strain for the inhibition adhesion assays. For the inhibition assays, three different treatments were followed: (i) bacterial cultures were added to Caco-2 monolayers simultaneously (competition); (ii) *Lactobacillus* spp. cells were added to Caco-2 monolayers 1 h before *L. monocytogenes* EGDe (exclusion); and (iii) *L. monocytogenes* EGDe cells were added to Caco-2 monolayers 1 h before *Lactobacillus* spp. (displacement). The plates were incubated for 1 h at 37 °C and 5% CO_2_ (CO2 Incubator CO2CELL 170 STD, Munich, Germany). Following incubation, each well was washed carefully twice with 500 μL phosphate buffered saline (PBS; TaKaRa, Kusatsu, Japan), and the adhered bacterial cells were harvested with 1 mL trypsin (Trypsin-EDTA 10X; Biosera Europe, Nuaillé, France). The *L. monocytogenes* EGDe cells were enumerated on *Listeria* Isolation Medium (Oxford Formulation) plates (LabM) after incubation for 48 h at 37 °C in aerobic conditions, and the lactobacilli were enumerated on MRS agar plates after 72 h at 37 °C under anaerobic conditions. The adhesion capacity of each bacterial strain was estimated as the number of adherent cells divided by the total cells added multiplied by 100. The inhibition of *L. monocytogenes* adhesion was calculated as follows: inhibition rate = 100(1 − T*_Lb_*/T), where T*_Lb_* and T are the numbers of adherent *L. monocytogenes* cells (CFU/well) in the presence and absence of lactobacilli, respectively. The experiments were performed in three biological and two technical replicates.

### 2.5. Invasion Assay

The inhibitory effect of lactobacilli against Caco-2 invasion by *L. monocytogenes* EGDe was estimated using the gentamicin-based assay according to Moroni et al. [24] and Zilelidou et al. [21]. Briefly, Caco-2 cell monolayers in 24-well plates were inoculated with *L. monocytogenes* EGDe or lactobacilli in single or dual cultures, as described above for the adhesion assays. The plates were incubated for 1 h at 37 °C and 5% CO_2_. The monolayers were washed twice with PBS to remove the non-adherent bacteria and then incubated in 0.5 mL/well DMEM with 0.1% FBS and 150 μg/mL gentamicin (Gentamicin sulfate, 10 mg/mL, Biosera Europe) for 45 min to kill the non-invaded *L. monocytogenes* EGDe cells. The Caco-2 monolayers were washed twice with 500 μL PBS and lysed with 1 mL ice-cold 0.1% (*v*/*v*) Triton™ X-100 (Fischer Scientific, Geel, Belgium). The population of the invaded *L. monocytogenes* EGDe cells were enumerated on TSA-YE after incubation for 48 h at 37 °C in aerobic conditions. The inhibition of invasion was calculated as follows: inhibition rate = 100(1 − T*_Lb_*/T), where T*_Lb_* and T are the numbers of invaded *L. monocytogenes* cells (CFU/well) in the presence and absence of lactobacilli, respectively. The experiments were performed in three biological and two technical replicates.

### 2.6. Statistical Analysis

The statistical analysis was performed using the SPSS Statistics for Windows version 16.0 (SPSS Inc., Chicago, IL, USA). For the pairwise comparison of the adhered bacterial populations on the Caco-2 cells at 1 h and 2 h, the Student’s *t*-test was used. For the comparison of the inhibition rates among the different strains, Tukey’s honestly significant differences (HSD) test was performed. The significance level was set at a *p*-value < 0.05.

## 3. Results

### 3.1. Antimicrobial Activity

The results of the antimicrobial assays are shown in Table 3. The spot-on-lawn assay showed that 21 out of 45 LAB strains were able to inhibit the growth of all ten *L. monocytogenes* strains by presenting an inhibitory zone around the lactobacilli spots. The most pronounced antilisterial effect was displayed by *L. rhamnosus* strains (LR-B19, LR-10, LR-B5, LR-B20, LR-A3, LA-A20, LR-C44, LR-52), *L. paragasseri* (LA-B17, LDD-C1), *L. crispatus* LC-C1 and the strain *L. gasseri* LG-C45. However, when the pH-neutralized CFS of the LAB strains were used in the agar-well diffusion assay, no growth inhibition of *L. monocytogenes* was observed.

### 3.2. Adhesion of Single Bacterial Strains on Caco-2 Cells

**Figure 1:**
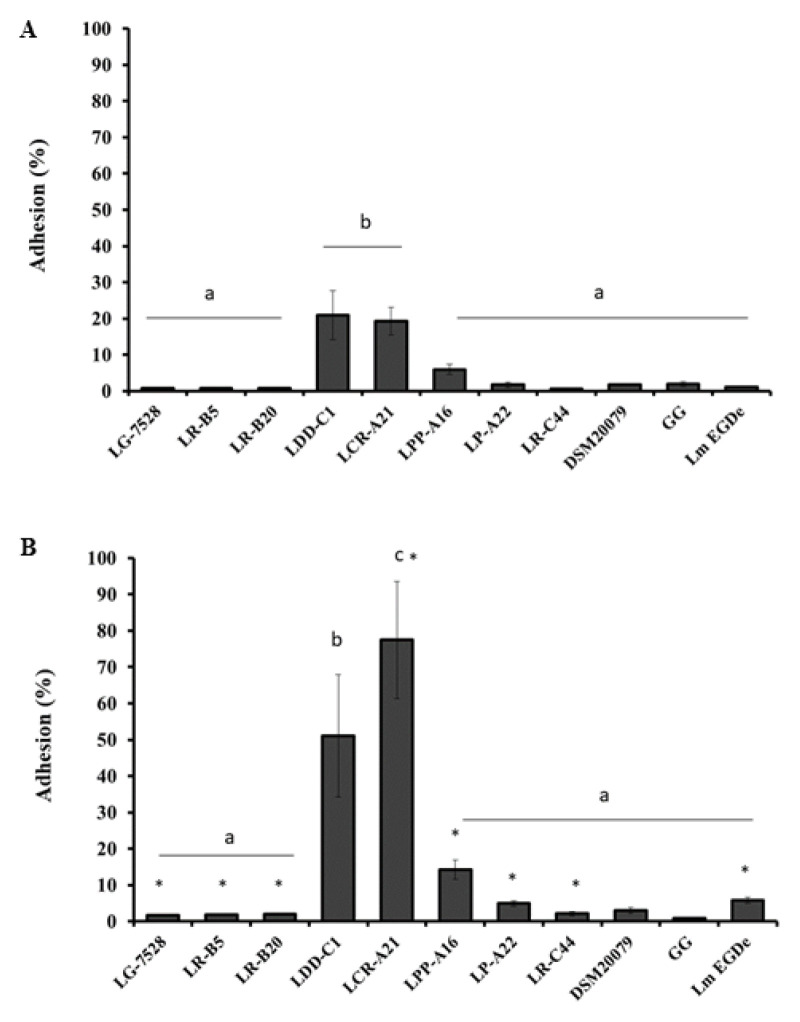
Adhesion efficiency (%) of the lactobacilli and *L. monocytogenes* strains to the Caco-2 cells following 1 h (**A**) and 2 h (**B**) of incubation. Data represent the mean values ± standard error of the mean (SEM) of three biological and two technical replicates. Different letters indicate significant differences among the strains (*p* < 0.05), and asterisks indicate significant differences between 1 h and 2 h (*p* < 0.05).

Following 1 h of contact of the single bacterial isolates with the Caco-2 cells, the adhesion efficiency of the lactobacilli ranged from 0.6% to 21% (Figure 1A). The best adhesion efficiency was observed for *L. paragasseri* LDD-C1 (21%) and *L. crispatus* LCR-A21 (19.3%), which were significantly higher compared to the reference strain *L. rhamnosus* GG (LGG, *p* < 0.05). *L. monocytogenes* EGDe adhered to the Caco-2 cells at a level of 1.1%, significantly lower compared to the aforementioned *Lactobacillus* strains (*p* < 0.05). Following 2 h of Caco-2 contamination, the numbers of adhered bacterial cells increased significantly (*p* < 0.05) and ranged from 0.8% to 77.5% (Figure 1B). Similar to 1 h, the best adhesion efficiency was observed for *L. paragasseri* LDD-C1 (51.1%) and *L. crispatus* LCR-A21 (77.5%). The strains *L. acidophilus* DSM20079 and LGG had no significant increase in adherence to Caco-2 during the 2 h of incubation.* L. monocytogenes* EGDe increased from 1.1% to 5.9% (*p* < 0.05).

### 3.3. Inhibition of Adhesion

**Figure 2:**
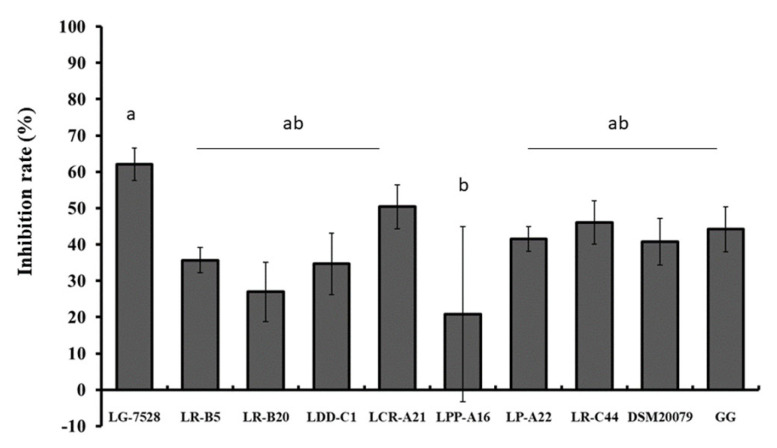
Inhibition (%) of *L. monocytogenes* EGDe adhesion to the Caco-2 cells by different lactobacilli strains in a competition assay, where the antagonistic bacteria were added to the epithelial cells at the same time. Data represent the mean values ± standard error of the mean (SEM) of three biological and two technical replicates. Different letters indicate significant differences among the strains (*p* < 0.05).

When *L. monocytogenes* and *Lactobaciillus* spp. isolates were added to the Caco-2 cells at the same time (competition), the inhibition of *L. monocytogenes* EGDe adherence ranged from 20.8% to 62.1% (Figure 2). The highest inhibition rates were observed for *L. gasseri* LG-7525 (62.1%), *L. crispatus* LCR-A21 (50.4%) and *L. rhamnosus* LR-C44 (46.1%).

**Figure 3:**
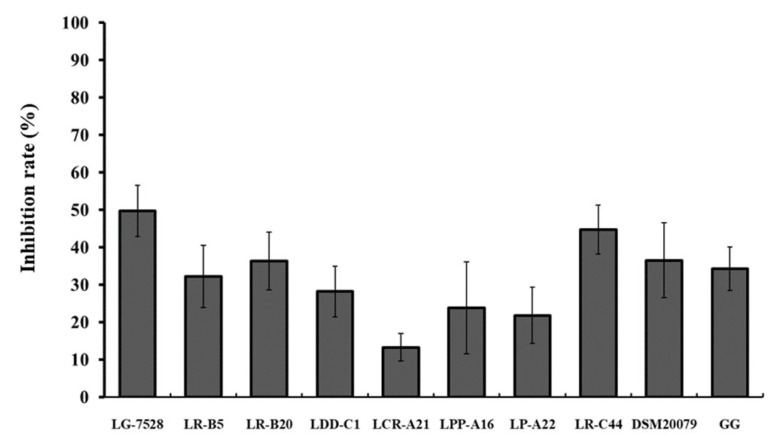
Effect of 1 h pre-incubation with lactobacilli strains on the *L. monocytogenes* EGDe adhesion to the Caco-2 cells (exclusion). Data represent the mean values ± standard error of the mean (SEM) of three biological and two technical replicates.

When the Caco-2 cells were pre-incubated for 1 h with *Lactobaciillus* spp. isolates before the addition of *L. monocytogenes* EGDe (exclusion), the inhibition rates ranged from 13.3% to 49.7% (Figure 3). The isolates *L. gasseri* LG-7528 (49.7%) and *L. rhamnosus* LR-C44 (44.7%) exhibited the highest rates of pathogen exclusion.

**Figure 4:**
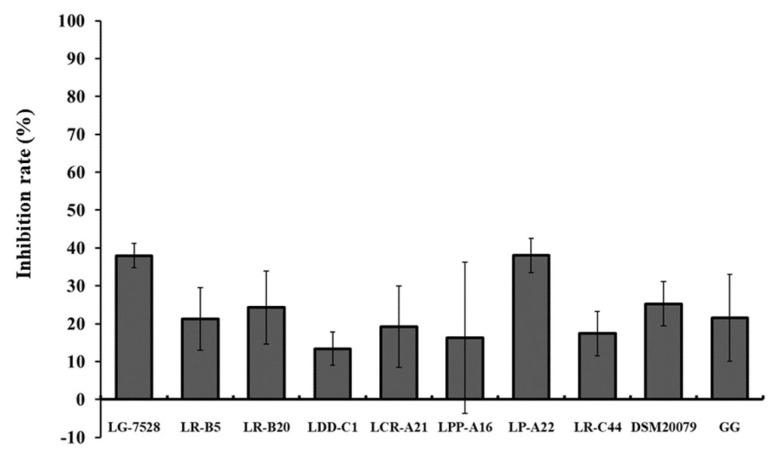
Inhibition (%) of *L. monocytogenes* EGDe adherence to the Caco-2 cells by lactobacilli strains after pre-incubation of the epithelial cells with the pathogen for 1 h (displacement). Data represent the mean values ± standard error of the mean (SEM) of three biological and two technical replicates.

In the displacement assay, where lactobacilli were added to the Caco-2 cells 1 h after contamination with *L. monocytogenes* EGDe, the adherence was limited by 13.4% to 38.0% (Figure 4). The greatest inhibition of *L. monocytogenes* adhesion (38.0%) was displayed by the isolates *L. gasseri* LG-7528 and *L. pentosus* LP-A22.

### 3.4. Inhibition of Invasion

**Figure 5:**
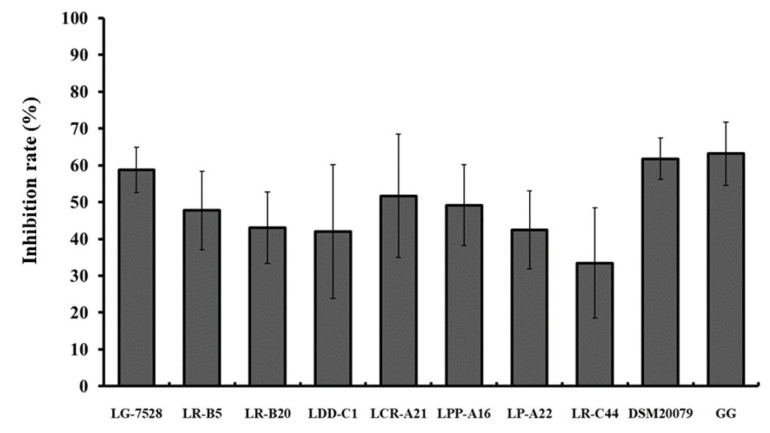
Effect of different lactobacilli strains on Caco-2 invasion by *L. monocytogenes* EGDe in a competition assay, where the antagonistic bacteria were added to the Caco-2 cells at the same time. Data represent the mean values ± standard error of the mean (SEM) of three biological and two technical replicates.

The inhibition of invasion of the Caco-2 cells by *L. monocytogenes* EGDe varied depending on the *Lactobacillus* spp. strain and the treatment method. In the competition assay, where *L. monocytogenes* EGDe and *Lactobacillus* spp. were added to the Caco-2 cells simultaneously, the invasion was reduced by 33.5% to 63.1% (Figure 5). The highest inhibition of invasion was recorded by the two reference strains, *L. acidophilus* DSM20079 (61.8%) and LGG (63.1%). Among the strains isolated from the neonatal feces,* L. gasseri* LG-7528 resulted in a decrease in invasion by 58.8%, *L. crispatus* LCR-A21 by 51.7%, *L. paracasei* subsp. *tolerans* LPP-A16 by 49.2% and *L. rhamnosus* LR-B5 by 47.7%.

**Figure 6:**
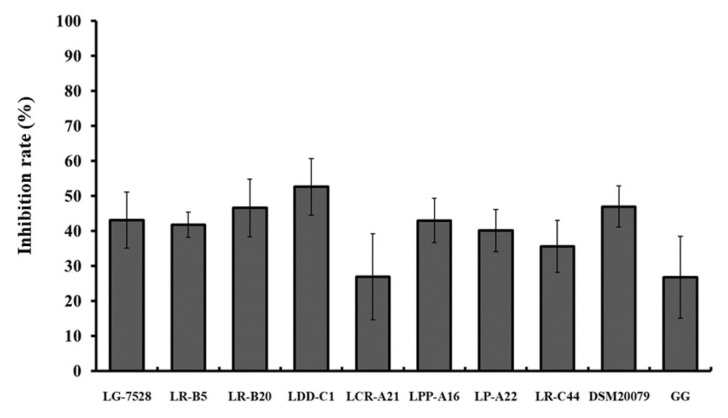
Effect of different lactobacilli strains on Caco-2 invasion by *L. monocytogenes* EGDe in an exclusion assay, where the Caco-2 cells were pre-treated with *Lactobacillus* spp. for 1 h before the addition of *L. monocytogenes* EGDe. Data represent the mean values ± standard error of the mean (SEM) of three biological and two technical replicates.

Pretreatment of the Caco-2 cells with lactobacilli for 1 h (exclusion assay) resulted in an inhibition of invasion at levels that ranged from 26.8% to 52.6%. The isolates *L. paragasseri* LDD-C1, *L. acidophilus* DSM20079 and *L. rhamnosus* LR-B20 were the most efficient in inhibiting *L. monocytogenes* EGDe to invade the Caco-2 cells at levels 52.6%, 47% and 46.6%, respectively (Figure 6).

**Figure 7:**
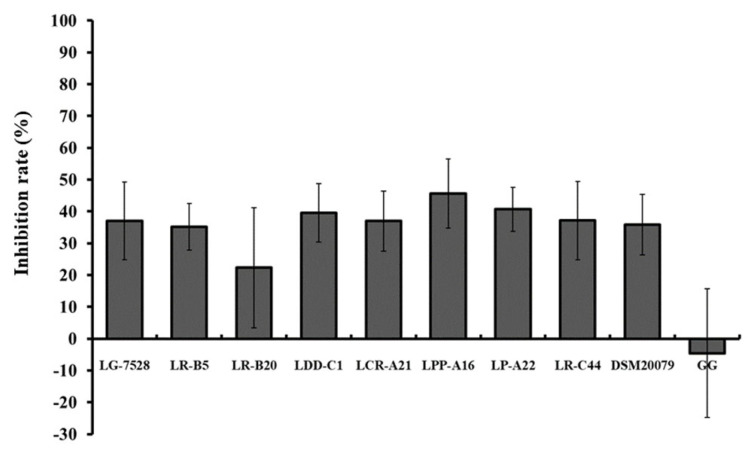
Effect of the presence of different lactobacilli strains on Caco-2 invasion by *L. monocytogenes* EGDe in a displacement assay, where the Caco-2 cells were pre-incubated with *L. monocytogenes* EGDe for 1 h before the addition of lactobacilli. Data represent the mean values ± standard error of the mean (SEM) of three biological and two technical replicates.

During the exclusion assay, where *Lactobacillus* spp. were added 1 h after contact of *L. monocytogenes* EGDe with Caco-2, the inhibition ranged from 22.3% to 45.6% (Figure 7). The highest displacement rates were observed for *L. paracasei* subsp.* tolerans* LPP-A16 (45.6%) and *L. pentosus* LP-A22 (40.6%). In the displacement assay, Caco-2 invasion by *L. monocytogenes* EGDe was increased by 4.5% with the addition of LGG.

## 4. Discussion

Lactic acid bacteria predominate the GI tract of healthy humans and confer numerous beneficial health effects, also providing protection against pathogens. In this study, we aimed to investigate lactobacilli strains previously isolated from the feces of healthy neonates for their inhibitory effect against the foodborne pathogen *L. monocytogenes*.

The antimicrobial activity against *L. monocytogenes* growth was estimated with two methods: the spot-on-lawn and the agar-well diffusion assays. In the latter, pH-neutralized cell-free supernatants were used to estimate the antimicrobial effect of lactobacilli metabolic substances. Antilisterial activity was observed with the spot-on-lawn method in contrast to the agar-well diffusion assay, where no CFS was able to inhibit *L. monocytogenes* growth. These findings are in accordance with other studies where pH-neutralized CFS presented limited antimicrobial activity compared to acid CFS [32]. Numerous studies have investigated the antimicrobial activity and effectiveness of lactic acid bacteria, and specifically lactobacilli, against food-borne pathogens [33,34,35]. The source of isolation, bacterial species and the methods used are factors that affect the estimation of antimicrobial activity of lactobacilli. In this study, antilisterial activity was mainly exhibited by the strains of the species *L. rhamnosus*. Syrokou et al. [36] investigated the antimicrobial activity of 207 LAB isolates and found that 23 strains were active against *L. monocytogenes* serotype 4b; however these strains belonged to the *L. plantarum* species, which was not included in our study, and were isolated from sourdough. Limited or no antimicrobial activity against *L. monocytogenes* was also observed for *L. paracasei* and *L. rhamnosus*, isolated from infant fecal samples, and *L. acidophilus*, isolated from pickled cabbage, examined with the agar-well diffusion method [37].

The ability of lactobacilli to adhere to epithelial cells is an important aspect of the probiotic that may confer a competitive advantage over pathogens for adhesion and invasion sites on epithelial cells. Among the ten *Lactobacillus* sp. strains tested for adherence to Caco-2 cells, the greatest adhesiveness was observed by *L. paragasseri* LDD-C1 and *L. crispatus* LCR-A21. The adhesion efficiency for these strains was greater than the reference strain LGG, which is considered highly adhesive [16,38,39,40]. The lower adhesive capacity of LGG compared to other commercial and potential probiotic bacteria has been previously reported [41]. *L. paragasseri* LDD-C1 and *L. crispatus* LCR-A21 were more adherent than *L. monocytogenes* EGDe. After 2 h of contact with the Caco-2 cells, the percentages of adhered bacteria increased significantly compared to 1 h for all the strains except *L. paragasseri* LDD-C1 and the two reference strains *L. acidophilus* DSM20079 and *L. rhamnosus* GG. The highest adhesion efficiency was achieved by *L. paragasseri* LDD-C1 and *L. crispatus* LCR-A21, while other lactobacilli and *L. monocytogenes* EGDe remained at low adherence levels. This is indicative of the good adhesive properties of the specific isolates. In a previous study, it was shown that incubation for 2 h and 4 h resulted in higher levels of adherence for *Salmonella enterica* but not for *L. paracasei*, showing that a longer contact time does not result in higher adhesion rates for all bacteria [15]. They also showed that the strongest inhibition of adhesion was observed for the shortest time of contact (2 h vs. 4 h).

The presence of lactobacilli reduced the adhesive ability of *L. monocytogenes* EGDe to the Caco-2 cells in a strain and treatment-dependent manner. The inhibition varied among the *Lactobacillus* strains, and there were isolates with greater blocking effect against pathogens compared to LGG. The results revealed that adhesion efficiency and inhibition of adhesion were two distinct events. For instance, *L. gasseri* LG-7528, a strain with low adhesion efficiency, exhibited the highest inhibitory capacity against *L. monocytogenes* EGDe in the competition and exclusion assays in contrast to the highly adhesive strains *L. paragasseri* LDD-C1, *L. crispatus* LCR-A21 and *L. paracasei* subsp. *tolerans* LPP-A16. Similar results were observed previously, where the low-adhesive lactobacilli strains were the most effective against *L. monocytogenes* [25,42].

Regarding the impact of treatment on the inhibition efficiency of lactobacilli, in the present study, inhibition was greater when the antagonistic bacteria were added to the Caco-2 cells simultaneously. The least inhibition rate was recorded in the displacement assay, where the lactobacilli were added to 1 h pre-contaminated Caco-2 cells with the pathogen. Previously, it has been suggested that the mechanisms of competition and exclusion are similar to each other and differ from displacement [25,43]. The displacement of GI bacteria including pathogens is probably a slow process with many of them needing 2 h to achieve increased degrees of displacement [43]. Pre-treatment of epithelial cells with probiotics was shown to result in increased inhibition of *L. monocytogenes* adherence, probably attributed to a mechanism related to co-aggregation of probiotic and pathogen cells [16]. Further studies are needed to elucidate these phenomena.

*L. monocytogenes* is an invasive pathogen and, once it is adhered to the eukaryotic cell surface, penetrates into the host cells beginning its intracellular lifecycle [44]. Probiotics that are able to block this internalization will be promising for a protective effect for human health. The presence of lactobacilli was able to reduce Caco-2 invasion by *L. monocytogenes* EGDe. The levels of inhibition varied among the different strains and treatments. Similar to the adherence process, competition resulted in a greater inhibition of invasion than exclusion and displacement. During competition, the reference strains *L. acidophilus* DSM20079 and *L. rhamnosus* GG were the most effective, resulting in higher inhibition rates than the other lactobacilli strains, followed by *L. gasseri* LG-7528. Although not highly adhesive, these isolates could effectively block Caco-2 invasion by the pathogen, thus demonstrating distinct mechanisms underlying these two processes. Moreover, the lactobacilli isolates exhibited different inhibitory effects in adhesion and invasion, which is in accordance with the results of Moroni et al. [24] who similarly reported different patterns by bifidobacterial strains against *L. monocytogenes*. Interestingly, the addition of LGG to the Caco-2 cells that were pre-contaminated with *L. monocytogenes* EGDe for 1 h increased the invasion of the pathogen in the epithelial cells. Similar results were observed in other studies in the process of adherence, where lactobacilli strains resulted in an increase in the adherence of pathogenic bacteria, such as *Clostridium difficile*, *Escherichia coli*, *L. monocytogenes* and *Salmonella* Typhimurium [16,25]. This could be of great concern; therefore, case-by-case studies on the interaction of potential probiotics with pathogenic bacteria should be conducted.

## 5. Conclusions

In conclusion, lactobacilli strains with probiotic properties isolated from the fecal samples of healthy neonates were able to inhibit *L. monocytogenes* adherence to and invasion in Caco-2 cells at variable levels. The inhibitory effect was strain and treatment dependent with competition resulting in greater inhibition compared to the exclusion and displacement assays. For each strain, the adhesive ability and inhibition of adhesion were distinct events, as well as the inhibition of adhesion and invasion. The results are indicative of the strain-specific properties of the lactobacilli; however, it is evident that some of these strains, including *L. gasseri* LG-7528, *L. paragasseri* LDD-C1, *L. crispatus* LCR-A21 and *L. paracasei* subsp.* tolerans* LPP-A16, could be further investigated for their potential probiotic use and their antimicrobial activity against other pathogens at growth, adherence and invasion in epithelial cells levels.

## Figures and Tables

**Table 1:** Lactic acid bacteria strains isolated from neonate fecal samples used in the study.

Isolate	Coding	Neonate Information
*Lacticaseibacillus rhamnosus*	LR-1	MCB + F
*Lactobacillus gasseri*	LG-7528	MCB + F
*Lactobacillus vaginalis*	LV-6	FCF
*Lacticaseibacillus rhamnosus*	LR-B19	MNB
*Lacticaseibacillus rhamnosus*	LR-10	FNB
*Lacticaseibacillus rhamnosus*	LR-A1	FNB
*Lacticaseibacillus rhamnosus*	LR-B5	MNB
*Lacticaseibacillus rhamnosus*	LR-B20	FCB
*Lactobacillus paragasseri*	LA-B17	FNB
*Lactobacillus acidophilus*	LA-B2	MNB
*Lactobacillus paragasseri*	LDD-C1	FCF
*Lactobacillus gasseri*	LA-A2	FNB
*Limosilactobacillus* sp.	LF-B15	FCB + F
*Lacticaseibacillus rhamnosus*	LR-A3	MNB + F
*Lacticaseibacillus rhamnosus*	LA-A20	FNB
*Limosilactobacillus fermentum*	LF-B14	FCB + F
*Lactobacillus crispatus*	LCR-A21	MNB + F
*Lactobacillus brevis*	LB-38	MNB
*Lactobacillus crispatus*	LC-40	FNB + F
*Lactobacillus salivarius*	LS-44	MNB
*Lacticaseibacillus rhamnosus*	LR-46	FCB + F
*Lacticaseibacillus paracasei* subsp. *tolerans*	LPP-A16	MNB
*Lactiplantibacillus pentosus*	LP-A22	MNB
*Enterococcus* sp.	E-49	FNB
*Lacticaseibacillus rhamnosus*	LR-51	FNB + F
*Lacticaseibacillus rhamnosus*	LR-52	FNB
*Lactobacillus crispatus*	LC-C1	FCB
*Lactobacillus gasseri*	LG-C5	MCB
*Lactobacillus gasseri*	LG-C9	FNB
*Lactobacillus gasseri*	LG-C15	FNB
*Lactobacillus gasseri*	LG-C28	FNB
*Lactobacillus gasseri*	LG-C32	FCB
*Lactobacillus gasseri*	LG-C39	MNB
*Lacticaseibacillus rhamnosus*	LR-C44	MNB
*Lactobacillus gasseri*	LG-C45	MNB
*Lactobacillus gasseri*	LG-C50	MNB
*Lactobacillus crispatus*	LC-C51	MNB
*Lacticaseibacillus rhamnosus*	LR-C58	MNB + F
*Lactobacillus gasseri*	LG-C59	MNB + F
*L. paracasei paracasei*	LPP-C68	MNB
*L. paracasei paracasei*	LPP-C70	FCB
*Lactobacillus gasseri*	LG-C72	FCB
*Lactobacillus gasseri*	LG-C74	FCB
*Lactobacillus acidophilus*	DSM20079	reference strain
*Lacticaseibacillus rhamnosus*	GG	reference strain

Abbreviations: F, Female; M, Male; N, Natural mode of delivery; C, Caesarean mode of delivery; B, Breast feeding exclusively; B + F, Breast and formula feeding; F, Formula feeding.

**Table 2:** *Listeria monocytogenes* strains used in the study.

Isolate	Origin	Year of Isolation	Country	Serotype
C5	Cow feces	2007	Ireland	4b
6179	Farmhouse cheese	1999	Ireland	1/2a
ScottA	Human isolate	1983	USA	4b
PL4	Dairy farm environment	2007	Greece	4b
PL11	Chicken	2007	Greece	1/2a
PL13	Chicken	2007	Greece	4b
PL18	Chicken	2007	Greece	1/2a
FL78	Meat	2012	Greece	4b
EGDe	Mammal	1924	[30]	1/2a
DSM12464	Mammal		[31]	1/2a

**Table 3:** Antimicrobial activity of the LAB isolates against the *L. monocytogenes* strains.

	*L. monocytogenes* Strains									
LAB Isolates	C5	6179	ScottA	PL4	PL11	PL13	PL18	FL78	EGDe	DSM12464
*L. rhamnosus* LR-1	-	+	+	+	+	+	+	+	+	+
*L. gasseri* LG-7528	-	+	+	+	+	+	+	+	+	+
*L. vaginalis* LV-6	-	-	-	-	-	-	-	-	-	-
*L. rhamnosus* LR-B19	++	+	++	++	++	++	++	+	+	+
*L. rhamnosus* LR-10	+	+	++	++	++	++	++	+	+	+
*L. rhamnosus* LR-A1	+	+	++	++	+	++	++	-	+	+
*L. rhamnosus* LR-B5	+	+	++	++	++	+++	++	+	+	+
*L. rhamnosus* LR-B20	+	+	++	++	++	++	++	+	+	++
*L. paragasseri* LA-B17	+	+	+	++	+	++	++	+	+	+
*L. acidophilus* LA-B2	++	++	+	+	+	-	+	+	-	+
*L. paragasseri* LDD-C1	+	+	++	++	++	++	+	+	+	+
*L. gasseri* LA-A2	+	++	++	++	+++	++	+	+	+	+
*Limosilactobacillus* sp. LF-B15	+	+	-	-	+	+	-	-		+
*L. rhamnosus* LR-A3	+	+	++	++	++	+++	+++	+	+	++
*L. rhamnosus* LA-A20	+	+	+	+	++	++	++	+	+	++
*L. fermentum* LF-B14	+	+	+	+	-	+	+	+	-	+
*L. crispatus* LCR-A21	+	+	++	++	+	++	+	+	+	+
*L. brevis* LB-38	+	-	-	-	-	-	-	-	-	-
*L. crispatus* LC-40	+	+	-	-	+	+	-	-	-	-
*L. salivarius* LS-44	++	++	+	+	+	+	+	+	+	+
*L. rhamnosus* LR-46	++	+	+	+	+	+	+	+	+	+
*L. paracasei* subsp. *tolerans* LPP-A16	++	++	+	+	+	+	+	+	+	+
*L. pentosus* LP-A22	++	++	+	+	+	+	+	+	-	+
*Enterococcus* sp. E-49	++	++	+	+	+	+	-	+	-	-
*L. rhamnosus* LR-51	++	++	+	+	+	+	+	+	+	+
*L. rhamnosus* LR-52	++	++	+	+	++	+	+	+	+	+
*L. crispatus* LC-C1	+	++	++	+	-	++	+	+	+	+
*L. gasseri* LG-C5	-	+	+	-	+	+	++	+	-	+
*L. gasseri* LG-C9	++	+	+	+	+	+	+	-	-	-
*L. gasseri* LG-C15	-	-	-	-	-	-	-	+	-	+
*L. gasseri* LG-C28	-	-	+	+	+	+	-	+	+	+
*L. gasseri* LG-C32	+	-	-	+	+	+	+	-	-	-
*L. gasseri* LG-C39	+	+	+	+	+	+	++	+	-	+
*L. rhamnosus* LR-C44	++	++	++	+	++	++	+	+	+	+
*L. gasseri* LG-C45	++	++	++	++	++	++	++	+	+	+
*L. gasseri* LG-C50	+	+	+	++	+	++	+	+	+	+
*L. crispatus* LC-C51	-	-	-	-	-	-	-	-	-	-
*L. rhamnosus* LR-C58	+	+	+	+	+	+	+	+	+	+
*L. gasseri* LG-C59	-	+	+	+	+	+	++	+	-	++
*L. paracasei paracasei* LPP-C68	-	-	+	-	+	+	+	+	-	+
*L. paracasei paracasei* LPP-C70	-	-	+	+	++	+	++	++	+	++
*L. gasseri* LG-C72	-	-	+	+	+	-	+	++	-	++
*L. gasseri* LG-C74	+	+	+	+	+	+	+	+	+	+
*L. acidophilus* DSM20079	+	+	+	+	+	+	+	+	+	+
*L. rhamnosus* GG	+	+	+	-	+	+	+	+	+	+

Symbols: -, no inhibition zone; +, inhibition zone up to 2 mm; ++, inhibition zone up to 4 mm; +++, inhibition zone over 6 mm.

## Data Availability

The data presented in this study are available on request from the corresponding author. The data are not publicly available due to privacy restrictions.
